# Enhanced fear memory after social defeat in mice is dependent on interleukin-1 receptor signaling in glutamatergic neurons

**DOI:** 10.1038/s41380-024-02456-1

**Published:** 2024-03-08

**Authors:** Ethan J. Goodman, Rebecca G. Biltz, Jonathan M. Packer, Damon J. DiSabato, Samuel P. Swanson, Braeden Oliver, Ning Quan, John F. Sheridan, Jonathan P. Godbout

**Affiliations:** 1 Department of Neuroscience, Wexner Medical Center, The Ohio State University, Columbus, OH USA; 2 Institute for Behavioral Medicine Research, College of Medicine, The Ohio State University, Columbus, OH USA; 3 Division of Biosciences, College of Dentistry, The Ohio State University, Columbus, OH USA; 4 Department of Biomedical Science, Brain Institute, Florida Atlantic University, Boca Raton, FL USA

**Keywords:** Neuroscience, Molecular biology

## Abstract

Chronic stress is associated with increased anxiety, cognitive deficits, and post-traumatic stress disorder. Repeated social defeat (RSD) in mice causes long-term stress-sensitization associated with increased microglia activation, monocyte accumulation, and enhanced interleukin (IL)-1 signaling in endothelia and neurons. With stress-sensitization, mice have amplified neuronal, immune, and behavioral responses to acute stress 24 days later. This is clinically relevant as it shares key aspects with post-traumatic stress disorder. The mechanisms underlying stress-sensitization are unclear, but enhanced fear memory may be critical. The purpose of this study was to determine the influence of microglia and IL-1R1 signaling in neurons in the development of sensitization and increased fear memory after RSD. Here, RSD accelerated fear acquisition, delayed fear extinction, and increased cued-based freezing at 0.5 day. The enhancement in contextual fear memory after RSD persisted 24 days later. Next, microglia were depleted with a CSF1R antagonist prior to RSD and several parameters were assessed. Microglia depletion blocked monocyte recruitment to the brain. Nonetheless, neuronal reactivity (pCREB) and *IL-1β* RNA expression in the hippocampus and enhanced fear memory after RSD were microglial-independent. Because *IL-1β* RNA was prominent in the hippocampus after RSD even with microglia depletion, IL-1R1 mediated signaling in glutamatergic neurons was assessed using neuronal *Vglut2*^*+*^*/*IL-1R1^−/−^ mice. RSD-induced neuronal reactivity (pCREB) in the hippocampus and enhancement in fear memory were dependent on neuronal IL-1R1 signaling. Furthermore, single-nuclei RNA sequencing (snRNAseq) showed that RSD influenced transcription in specific hippocampal neurons (DG neurons, CA2/3, CA1 neurons) associated with glutamate signaling, inflammation and synaptic plasticity, which were neuronal IL-1R1-dependent. Furthermore, snRNAseq data provided evidence that RSD increased CREB, BDNF, and calcium signaling in DG neurons in an IL-1R1-dependent manner. Collectively, increased IL-1R1-mediated signaling (monocytes/microglia independent) in glutamatergic neurons after RSD enhanced neuronal reactivity and fear memory.

## Introduction

Psychosocial stress is associated with increased anxiety and depression [1]. Chronic or traumatic stressors are linked with stress-sensitization, which is represented by an enhanced vulnerability and reactivity to subsequent stressors [2, 3]. Post-traumatic stress disorder (PTSD) is a manifestation of stress-sensitization and affects about 6% of the US population [4]. Repeated social defeat (RSD) in mice promotes the convergence of neuronal, central inflammatory, and peripheral immune pathways causing prolonged anxiety, social avoidance, and stress-sensitization [5–7]. Stress-sensitization after RSD persists 24–30 days later [5–7] and results in amplified responses to RSD re-exposure promoting inflammation, neuronal reactivity, and behavioral deficits (e.g., anxiety, social-withdrawal, cognitive-impairment) [8]. This enhanced stress reactivity after RSD is clinically relevant and shares key elements with PTSD [8].

A notable feature of this stress-sensitization is that anxiety recurs with re-exposure to acute defeat (1 day of RSD). By 24 days, stress-associated anxiety, splenomegaly, circulating cytokines, myelopoiesis, and monocyte accumulation in the brain has resolved. Nonetheless, several indices of sensitization persist at 24 days including social avoidance of an aggressive intruder [5], altered transcriptional profiles of microglia [7] and an increased reservoir of monocytes in the spleen [9]. Stress-sensitization is associated with the recurrence of inflammatory Ly6C^hi^ monocytes in circulation and in the brain, neuroinflammation, and anxiety in the open-field with RSD re-exposure [5–7, 10]. In the hippocampus, dentate gyrus (DG) neurons from stress-sensitized mice have functional differences with re-exposure to stress at D24 (acute defeat) with increased neuronal phospho-cAMP-response element binding protein (pCREB) induction compared to naïve mice [7]. While our work has focused on microglia, monocyte, and endothelia interactions in stress-induced anxiety [11–14], the goal of this study was to understand the influence of social defeat on hippocampal neurons in the context of enhanced fear memory.

Stress induces neuronal activation within regions of fear and threat appraisal in the brain (e.g., pre-frontal cortex, hippocampus, amygdala) [12, 13, 15]. Neurons from these regions in stress-sensitized mice have functional differences with re-exposure to stress at 24 days with increased pCREB induction compared to naïve controls [7, 10]. Increased expression of pCREB is implicated in learning-induced synaptic plasticity and may indicate increased neuronal reactivity to threatening stimuli [10, 16, 17]. This is clinically relevant because individuals diagnosed with PTSD have abnormal neuronal transmission associated with an overactive amygdala and reduction in hippocampus volume [18, 19]. Consistent with neuronal sensitization, the interpretation of fear was enhanced after RSD [20]. For example, mice had enhanced contextual fear memory (hippocampal-dependent) 8 days after RSD. Thus, stress causes sensitization in neurons, especially in the hippocampus, resulting in exaggerated responses to subthreshold stressors.

Stress-induced IL-1 signaling uses endothelial IL-1 receptor-1 (IL-1R1) and neuronal IL-1R1 [21]. Microglia/monocyte signaling to endothelia IL-1R1 elicits anxiety-like behavior [12, 14, 22] and neuronal IL-1R1 mediates social withdrawal and cognitive impairment [23]. While myeloid cells release IL-1β with stress, other cells in the CNS may also produce IL-1β. Even a low level of IL-1 release has a profound effect on neurons [24]. This is because IL-1R1 and corresponding accessory proteins are expressed on neurons in the cortex, hippocampus, and brainstem [21, 25]. Both IL-1β and IL-1 receptor antagonist (RA) bind to IL-1R1 [26, 27]. Moreover, there is robust IL-1R1 expression on excitatory glutamatergic (*Vglut2*^*+*^) neurons of the hippocampus [21, 23]. As such, IL-1 activation increases glutamate signaling in the brain [28]. IL-1 signaling in neurons, especially DG (*Vglut2*^*+*^) neurons, has an important role in the sensitization and re-activation of neurons during stress re-exposure to promote fear memory. Blockade of neuronal IL-1R1 signaling prevented key neuronal aspects in the establishment of stress-sensitization [10]. Additionally, pCREB reactivity and acute cognitive impairments were evident after acute defeat in stress-sensitized mice, but social withdrawal was blocked by IL-1RA [10]. Thus, neuronal IL-1R1 plays an important role in sensitizing neurons following RSD.

Our recent data indicate neuronal sensitization involved increased IL-1R1 signaling specifically in excitatory neurons of the hippocampus [10, 23]. Thus, the goal here was to investigate the influence of stress on hippocampal neurons in the context of enhanced fear memory and determine the degree to which microglia and IL-1R1 pathways were involved. Here we show novel data that cell and region-specific IL-1 (*Vglut2*^*+*^/IL-1R1) signaling mediated fear memory following RSD. Additionally, snRNAseq demonstrated that stress-induced pathways associated with neuronal transmission and synaptic plasticity, dependent on neuronal IL-1R1.

## Methods

### Mice

Male C57BL/6 (5–7 weeks) and CD-1 aggressors were purchased from Charles River Laboratories. IL-1R1^+/+^ (Cre^−^) and *Vglut2*^*+*^/IL-1R1^−/−^ (Cre^+^) mouse lines were bred in-house as described [29–32]. All procedures were in accordance with NIH Guidelines and the OSU Institutional Laboratory Animal Care and Use Committee.

### Repeated social defeat (RSD)

Mice were subjected to RSD as described [15]. In brief, a male CD-1 aggressor mouse was placed into the home cage of experimental mice (3 male mice/cage) for 2 h (16:00–18:00) per night for six consecutive nights. Control mice were left undisturbed.

### Plexxikon(PLX)-5622

PLX5622 was formulated in AIN-76A rodent chow (1200 mg/kg) [33]. Standard AIN-76A diet was provided as vehicle. PLX5622 or vehicle diets were provided for 7 days to deplete microglia prior to RSD [34].

### Fear conditioning

For the acquisition trial, mice were habituated for 90 s followed by a 30 s (2000 Hz, 70 db) tone. A 0.5 mA shock co-terminated during the last 2 s. The tone/shock was repeated five times (30 s inter-trial-interval (ITI)). For the contextual trial (24 h later), mice were habituated in the behavioral suite for 45 min then percent freezing was recorded for 10 min (context A) [35]. For the cued trial (24 h later), mice were habituated for 90 s in a new context (context B) with checkered floors/walls and banana extract. A tone played five times (30 s ITIs). % freezing during the tones was quantified. Each cohort was randomized, and experimenters were blinded to the treatments. Data were analyzed using Fusion software.

### PCREB, IBA1, and CD45 detection

Immunohistochemical analyses were completed as described [36]. In brief, brains were post-fixed, cryoprotected, and sectioned (30 µm). Sections were washed, blocked, and incubated with primary antibodies: anti-CD45, anti-IBA1, or anti-pCREB overnight. Separate sections were used for each label. Next, sections were washed and incubated with fluorochrome-conjugated secondary antibodies. Sections were washed, mounted on slides, and imaged using an EVOS M7000 system. pCREB sections were counterstained with DAPI. Percent area or mean fluorescent intensity were assessed using ImageJ. Experimenters were blinded to the treatments during image capture and analysis.

### RNA isolation and qPCR

Total RNA was extracted from the hippocampus using Tri-Reagent and cDNA was generated from the High-Capacity Reverse Transcription Kit. Quantitative real-time PCR was completed with TaqMan Gene Expression assay. Fluorescence was determined using QuantStudio3 Real-Time PCR System.

### In situ hybridization of IL-1β

RNAscope was performed as described [37]. In brief, sections were washed, heated, post-fixed, and dehydrated in ethanol. Antigens were retrieved with H_2_O_2_. Next, sections were treated with Protease-III for 30 min followed by IL-1β-C1 probe hybridization. Probe Amplification was completed, and signal was developed with Opal-690 dye. Images were captured using a Leica SP8 confocal (63X). Percent area of *IL-1β* RNA was quantified using ImageJ. With ×63 magnification only the granule cell layer of the DG was quantified. Experimenters were blinded to the treatments during each step.

### Nuclei isolation

Hippocampi (*n* = 3) were extracted and pooled. Pooled samples were homogenized to release nuclei and incubated with Myelin Removal Beads-II. Samples were filtered through LS columns, clarified, and washed. Nuclei were counted, fixed with a Nuclei Fixation Kit (Parse Biosciences), and frozen at −80 °C.

### Single-nuclei barcoding/sub-library generation

Parse Biosciences Whole Transcription Kit was used to barcode and generate sub-libraries with 12,500 nuclei/sub-library comprised of all samples. The resulting cDNA was sequenced at 40,000 reads/nuclei using a NovaSeq S4.

### Data processing

Fastq.gz files were aligned to Genome Reference Consortium Mouse Reference 39 using the Parse Biosciences pipeline. Matrices were filtered in RStudio using Seurat (v4.1.1) [38]. Nuclei with >20% mitochondrial DNA were excluded. After clustering, Uniform Manifold Approximation and Projection (UMAP), annotation was performed using established markers [34, 39–43]. Syt1^+^ neurons were subclustered and differential gene expression was performed using the FindMarkers with Model-based Analysis of Single-cell Transcriptomics (MAST) [44]. Pathway, regulators, and gene ontology (GO) analyses were performed with Ingenuity Pathway Analysis (IPA;Qiagen) [45] and Metascape [46].

### Statistical analysis

Data was analyzed GraphPad Prism 9 with *T*-tests (two-sided) or two-way ANOVAs to determine main effects and interactions. Tukey HSD was used for post hoc analysis when main effects or interactions were significant.

## Results

### Stress enhanced fear memory acutely and chronically

**Fig. 1:**
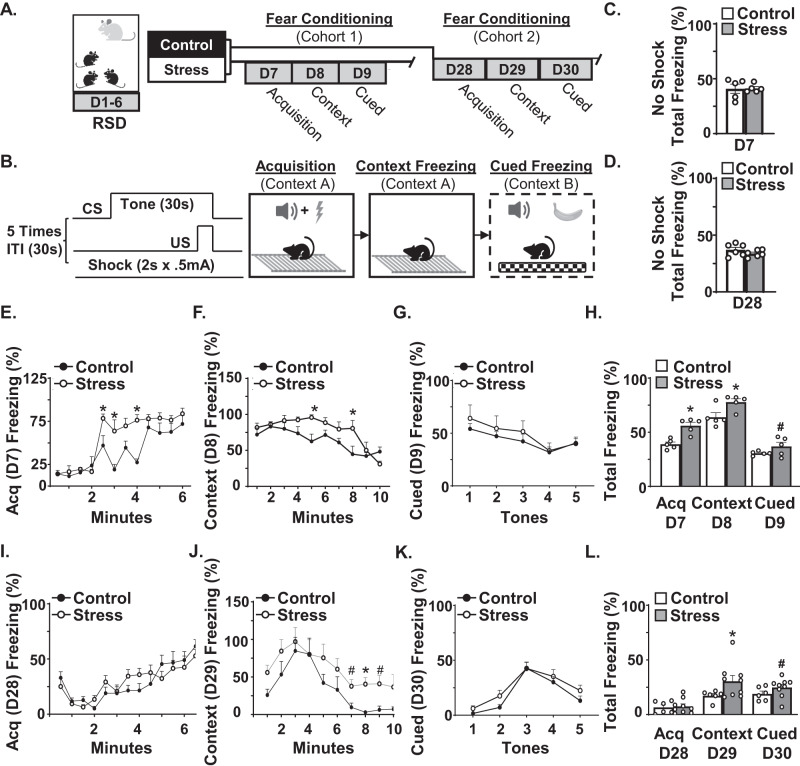
Stress enhanced fear memory acutely and chronically. **A** Male C57BL/6 mice were subjected to repeated social defeat (stress) or were undisturbed (control). Next, mice were exposed to the fear conditioning paradigm 1 day or 22 days later. **B** The fear conditioning paradigm consisted of 5 shocks (2 s × 0.5 mA) which co-terminated with the last 2 s of a 30 s tone. For the acquisition trial, the tone/shock was repeated five times in context A and freezing was determined (10 min). For the contextual trial, mice were placed in the same environment (context A) and freezing was determined (10 min). For the cued trial, mice were exposed to the tone protocol (five tones without shocks) in a novel context with banana scent and checkered flooring and walls (context B). First, a study was completed to confirm there were no differences in total percent time freezing between control and stress (*n* = 5). **C** Baseline total freezing determined in the absence of shock on D7. **D** Baseline total freezing on D28 in the absence of shock. **E** In the first cohort (*n* = 6), percent time freezing over 6 min in the fear acquisition (D7) trial (*F*(1,96) = 33.1, *p* < 0.001). **F** Percent time freezing over 10 min during contextual fear (D8) trial (*F*(1,80) = 15.97, *p* < 0.0001). **G** Percent time freezing after each tone during cued fear trial (*p* = 0.1). **H** Total time spent freezing during each trial (acquisition, context, and cued). **I** In the second cohort (*n* = 9), percent time freezing over 6 min in the fear acquisition (D28) trial. **J** Percent time freezing over 10 min in the contextual fear (D29) trial (*F*(1,130) = 15.95, *p* < 0.0001). **K** Percent time freezing after each tone during cued fear (D30) trial. **L** Total percent time spent freezing during each trial (acquisition, context, and cued). Graphs represent the mean ± SEM, and individual data points are provided. Means with (*) are significantly different from controls (*p* < 0.05) and means with (#) tend to be different from controls (*p* = 0.1).

RSD causes sensitization of neurons [29]. The mechanisms underlying stress-sensitization are unclear, but enhanced fear memory may be critical. Therefore, we aimed to determine the influence of microglia and neuronal IL-1R1 signaling in the development of stress-sensitization and RSD-induced fear memory. We assessed if stress influenced fear memory acutely (D7) and chronically (D28) using a fear conditioning paradigm (Fig. 1A, B). First, we confirmed there were no differences in total percent freezing between control and stress without the shock at D7 (Fig. 1C) or D28 (Fig. 1D).

Next, we assessed if stress influenced fear memory acutely (D7) using cohort 1. During acquisition (D7), there was a main effect of stress on the percent freezing (*F*(1,96) = 33.1, *p* < 0.001; Fig. 1E). Post hoc analysis confirmed that stress increased freezing compared to controls at several timepoints (*p* < 0.05). Moreover, stress increased percent total freezing during fear acquisition (*p* < 0.05, Fig. 1H). During the contextual trial (D8), there was a main effect of stress on the percent freezing (*F*(1,80) = 15.97, *p* < 0.0001; Fig. 1F). Post hoc analysis confirmed that stress increased freezing compared to controls at several timepoints (*p* < 0.05). Moreover, the percent total freezing during contextual fear (D8) was highest in the stress group (*p* < 0.0001; Fig. 1H). There were modest effects of stress during the cued trial (D9). Total percent freezing tended to be highest in the stress group compared to all other groups (*p* = 0.1; Fig. 1G, H).

Fear conditioning was next assessed on D28 (22 days after RSD) in cohort 2. There was no effect of stress on acquisition on D28 (Fig. 1I, L). Notably, the total percent freezing during acquisition (D28) was lower than non-shocked mice in Fig. 1D. Nonetheless different mice were used for Fig. 1D, L. During the contextual trial (D29), there was a main effect of stress on percent freezing (*F*(1,130) = 15.95, *p* < 0.0001; Fig. 1J). Post hoc analysis confirmed that stress increased freezing during the contextual trial compared to controls at several timepoints (*p* < 0.05). Moreover, the percent total percent freezing during the contextual fear trial (D29) was highest in the stress group (*p* < 0.05; Fig. 1L). For cued fear (Fig. 1K, L), only percent freezing during the tone presentation is shown. There were limited effects of stress during cued fear (D30) (Fig. 1K). The total percent cued freezing tended to be highest in the stress group compared to controls (*p* = 0.1; Fig. 1K). Collectively, stress enhanced fear memory acutely and contextual fear memory persisted weeks later.

### Stress-induced fear memory, pCREB activation and IL-1β expression in the hippocampus were microglia independent

**Fig. 2:**
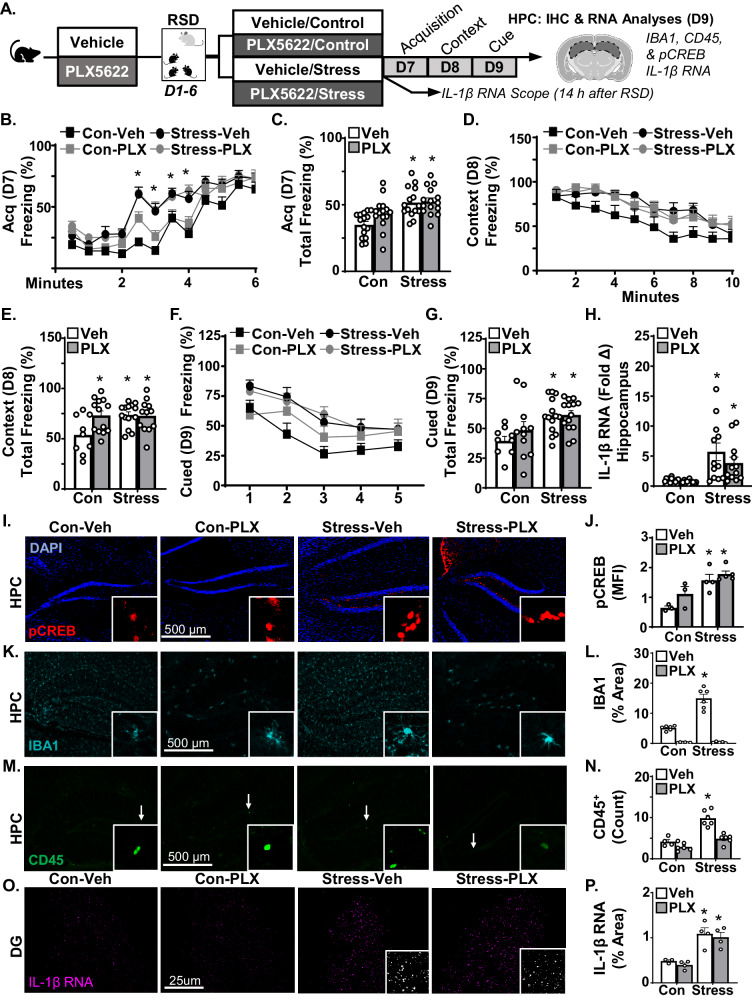
Stress-induced fear memory, pCREB activation and IL-1β expression in the hippocampus were microglia independent. **A** Male C57BL/6 mice were provided diets formulated with vehicle (Veh) or PLX5622 for 7 days. Next, mice were subjected to RSD (stress) or left undisturbed (control) and exposed to the fear conditioning paradigm 1 day later (*n* = 15). Mice were maintained on vehicle or PLX5622 diet for the study duration (16 days). **B** Percent time freezing over 6 min (*F*(3,672) = 38.4, *p* < 0.0001) and (**C**) total time freezing in the fear acquisition (D7) trial (*F*(1,56) = 21.6, *p* < 0.0001). **D** Percent time freezing over 10 min (*F*(3,430) = 13.2, *p* < 0.0001) and (**E**) total time freezing during contextual fear (D8) trial (*F*(1,44) = 4.5, *p* < 0.05). **F** Percent time freezing after each tone (*F*(3,210) = 9.1, *p* < 0.0001) and (**G**) total time freezing in the cued fear (D9) trial (*F*(1,44) = 12.4, *p* < 0.002). Immediately after cued fear testing on D9, mice were sacrificed and brains were collected for analyses. For RT-qPCR, hippocampi were microdissected and RNA was isolated (*n* = 11–12). **H** *IL-1β* mRNA in the hippocampus (*F*(1,43) = 18.98, *p* < 0.0001). For IHC samples, brains were fixed, sectioned, and labeled. **I** Representative labeling of pCREB and (**J**) Mean fluorescent intensity (MFI) of pCREB (*n* = 3–5) in the DG of the hippocampus relative to control (*F*(1,12) = 6.7, *p* < 0.05). **K** Representative labeling of IBA1 and (**L**) percent area of IBA1 labeling in the hippocampus (*F*(1,19) = 41.73, *p* < 0.0001). **M** Representative labeling of CD45^+^ and (**N**) number of CD45^+^ cells (*n* = 4–6) in the hippocampus (*F*(1,18) = 10.3, *p* < 0.005). In a separate experiment, RNA levels of *IL-1β* were determined in the DG 14 h after RSD using RNAscope (*n* = 3–4). **O** Representative labeling of *IL-1β* RNA in the DG (63x). **P** Percent area of *IL-1β* RNA in the DG (*F*(1,11) = 37.3, *p* < 0.0001). Graphs represent the mean ± SEM, and individual data points are provided. Means with (*) are significantly different from controls (*p* < 0.05).

Microglia activation, monocyte recruitment, and IL-1 expression are key elements in stress-induced anxiety in mice [6]. To assess the role of microglia/monocytes in enhanced fear memory, mice were administered vehicle or CSFR1 antagonist (PLX5622) diet to deplete microglia 1 week prior to RSD. These mice were maintained on experimental diets for the study duration. Fear conditioning was assessed on D7-9 and samples were collected for analyses following cued fear on D9 (Fig. 2A).

As expected, stress increased the percent freezing (*F*(3,672) = 38.4, *p* < 0.0001; Fig. 2B) and total percent freezing (*F*(1,56) = 21.6, *p* < 0.0001; Fig. 2C) during acquisition (D7). This effect of stress, however, was independent of microglia depletion. For contextual fear conditioning (D8), stress increased percent freezing (*F*(3,430) = 13.2, *p* < 0.0001; Fig. 2D) and total percent freezing (*F*(1,44) = 4.5, *p* < 0.04; Fig. 2E). These effects were independent of microglia depletion. During cued fear (D9), stress increased percent freezing (*F*(3,210) = 9.1, *p* < 0.0001; Fig. 2F) and total percent freezing (*F*(1,44) = 12.4, *p* < 0.002; Fig. 2G). These effects on cued fear memory were independent of microglia depletion. Thus, fear memory after stress was independent of monocytes/microglia.

Stress-sensitization after RSD is associated with neuronal reactivity with enhanced pCREB activation in the pre-frontal cortex and hippocampus after exposure to an acute stressor [7, 29]. In this design, the acute stressor is exposure to fear conditioning. Here, the influence of stress and PLX5622 on pCREB activation was assessed in the DG granule layer immediately after cued fear testing (D9). pCREB was increased by stress (*F*(1,12) = 6.7, *p* < 0.03, Fig. 2I, J) and post hoc analysis (*p* < 0.05) confirmed that the highest pCREB activation was in stress-sensitized mice exposed to fear conditioning (Stress-Veh and Stress-PLX). Moreover, this increased pCREB in the hippocampus in stress-sensitized mice was independent of microglia. Thus, enhanced pCREB activation in the hippocampus with stress-sensitization was microglia/monocyte independent.

Next, the influence of RSD and PLX5622 on IL-1β RNA, microglial proportional area (IBA1^+^), monocyte accumulation (CD45^+^ cells) was determined in the hippocampus after RSD and fear conditioning (D9). First, IL-1*β* RNA was increased in the hippocampus after stress. This increase was unaffected by microglia depletion (*F*(1,43) = 18.98, *p* < 0.0001, Fig. 2H). PLX5622 reduced IBA1^+^ % area in the hippocampus (*F*(1,19) = 41.73, *p* < 0.0001; Fig. 2K, L). These data are consistent with microglia depletion. Stress also increased % area of IBA1^+^ in the hippocampus (*F*(1,19) = 166.9, *p* < 0.0001). CD45^+^ cells (monocytes) were increased after stress (*F*(1,18) = 43.0, *p* < 0.0001) and was prevented by microglia depletion (*F*(1,18) = 10.3, *p* < 0.005, Fig. 2M, N). These data are consistent with previous RSD studies of microglia depletion [7, 12].

In a separate study, the influence of stress and PLX5622 on *IL-1β* RNA in the DG was assessed by RNAscope 14 h after RSD. Stress increased *IL-1β* RNA in the DG (*F*(1,11) = 37.3, *p* < 0.0001), but same as above, it was independent of microglia (Fig. 2O, P). Thus, increased *IL-1β* RNA after RSD in the DG was monocyte/microglia independent.

### Stress-induced fear memory and pCREB activation in the hippocampus were dependent on neuronal IL-1R1

**Fig. 3:**
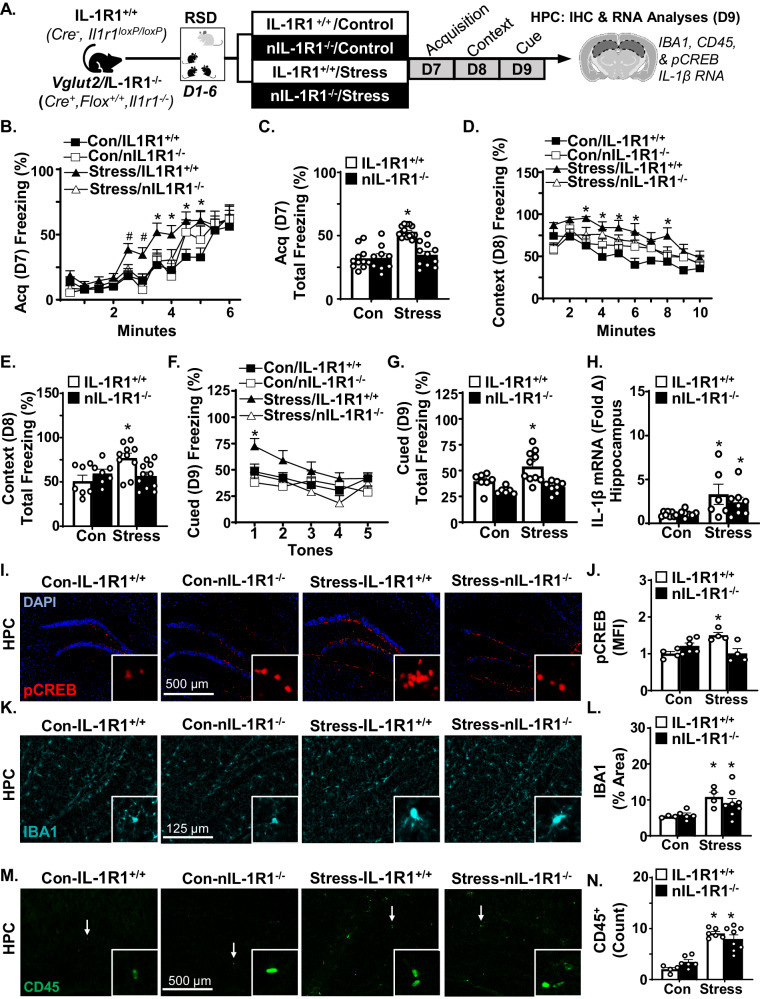
Stress-induced fear memory and pCREB activation in the hippocampus were dependent on neuronal IL-1R1. **A** Male IL-1R1^+/+^ and *Vglut2*^*+*^/IL-1R1^−/−^ (nIL-1R1^−/−^) mice were subjected to RSD or were undisturbed (control). Next, mice were exposed to the fear conditioning paradigm 1 day after RSD (*n* = 5). **B** Percent time freezing (*F*(3,502) = 16.30, *p* < 0.0001) and (**C**) total time freezing over 6 min in the fear acquisition (D7) trial (*F*(1,42) = 15.19, *p* < 0.0001). **D** Percent time freezing (*F*(3,280) = 19.53, *p* < 0.0001) and (**E**) total time freezing over 10 min in the contextual fear (D8) trial (*F*(1,31) = 4.62, *p* < 0.05). **F** Percent time freezing (*F*(3,140) = 8.998, *p* < 0.0001) and (**G**) total time spent freezing after each tone in the cued fear (D9) trial (*F*(1,27) = 6.03, *p* < 0.0001). Immediately after cued fear testing on D9, mice were sacrificed, and brains were collected. For RT-qPCR, hippocampi were microdissected and RNA was isolated (*n* = 6–8). **H** *IL-1β mRNA* in the hippocampus (*F*(1,25) = 9.5, *p* < 0.006; **H**). For IHC, samples were fixed, sectioned, and labeled. **I** Representative images of pCREB labeling and (**J**) mean fluorescent intensity (MFI) of pCREB in labeling the DG of the hippocampus (*n* = 3–5) relative to control (*F*(1,14) = 19.98, *p* < 0.001). **K** Representative images (*n* = 3–8) of IBA1 labeling and (**L**) % area of IBA1 labeling in the hippocampus (*F*(1,16) = 10.5, *p* < 0.005). **M** Representative CD45^+^ labeling and (**N**) number of CD45^+^ cells in hippocampus, amygdala, and pre-frontal cortex combined (*n* = 4–8) (*F*(1,20) = 75.9, *p* < 0.0001). Graphs represent the mean ± SEM, and individual data points are provided. Means with (*) are significantly different from controls (*p* < 0.05).

We show that enhanced fear memory after RSD was microglia/monocyte independent, but still associated with increased *IL-1β* RNA in the hippocampus/DG. This is relevant because IL-1R1 is highly expressed on excitatory neurons in the DG [30] and this pathway is important for stress-sensitization [29, 47]. Our lab and others have used pCREB labeling to assess neuronal re-activity to secondary stressors after mice have been stress sensitized by RSD [7, 48]. Thus, neuronal *Vglut2*^+^/IL-1R1^−/−^ (nIL-1R1^−/−^) mice were used to determine if enhanced fear memory following RSD was dependent on IL-1 receptor signaling in hippocampal excitatory neurons. Fear conditioning was assessed on D7–9 after RSD and samples were collected for analyses following cued fear on D9 (Fig. 3A).

Fear conditioning was assessed in IL-1R1^+/+^ and nIL-1R1^−/−^ mice acutely (D7–9). As in Fig. 1, stress increased the percent total freezing during acquisition (*F*(1,42) = 15.19, *p* < 0.0001), context (*F*(1,31) = 4.624, *p* < 0.05) and cued (*F*(1,27) = 6.03, *p* < 0.0001; Fig. 3B–G). These enhancements were influenced by neuronal IL-1R1 signaling. For instance, stress increased percent freezing (*F*(3,502) = 16.30, *p* < 0.0001; Fig. 3B) and total percent freezing (*F*(1,42) = 15.19, *p* < 0.05; Fig. 3C) during acquisition (D7) which was nIL-1R1 dependent. Post hoc analysis confirmed that percent total freezing was highest in Stress-IL-1R1^+/+^ mice compared to all other groups including Stress-nIL-1R1^−/−^ mice (*p* < 0.05, Fig. 3C). This interaction was also evident (*F*(3,280) = 19.53, *p* < 0.0001) during context fear (D8). The effects of stress on the percent total freezing (*F*(1, 31) = 7.2, *p* < 0.05; Fig. 3D) were dependent on nIL-1R1. Post hoc analysis confirmed that the Stress-IL-1R1^+/+^ group had the highest total percent freezing compared to all other groups (*p* < 0.05; Fig. 3D). During cued fear (D9), stress increased the percent total freezing (*F*(3,140) = 8.9, *p* < 0.0001; Fig. 3F) and total percent freezing (*F*(1,27) = 6.03, *p* < 0.05; Fig. 3G). Post hoc analysis confirmed that Stress-IL-1R1^+/+^ was increased compared to all other groups (*p* < 0.05). Together, stress enhanced fear memory via neuronal IL-1R1 signaling in the DG.

Next, the influence of RSD, fear conditioning, and nIL-1R1 on IL-1β RNA, pCREB^+^ activation, IBA1^+^ proportional area, and monocyte accumulation (CD45^+^) were determined in the hippocampus. As expected, stress increased *IL-1β* RNA levels in the hippocampus (*F*(1,25) = 9.5, *p* < 0.006; Fig. 3H) independent of IL-1R1 signaling. RSD-induced pCREB in DG (*F*(1,14) = 6.7, *p* < 0.03 Fig. 3H, I) and this increase was dependent on nIL-1R1 (*F*(1,14) = 19.9, *p* < 0.001; Fig. 3H, I). The Stress-IL-1R1^+/+^ group had the highest levels of pCREB compared to all other groups including the Stress-nIL-1R1^−/−^ group (*p* < 0.05). IBA1^+^ proportional area (*F*(1,16) = 10.5, *p* < 0.005; Fig. 3K, L) and number of CD45^+^ monocytes (*F*(1,20) = 75.9, *p* < 0.0001; Fig. 3M, N) were increased after RSD independent of nIL-1R1 [47]. Collectively, IL-1R1 signaling in *Vglut2*+ neurons mediated the stress-induced enhancement in fear memory and pCREB^+^ activation in the hippocampus.

### Stress and nIL-1R1 knockout influence single-nuclei RNAseq clustering and profiles

We show that stress enhanced fear memory and pCREB activation in the hippocampus, dependent on IL-1R1, but microglia/monocyte independent. To determine transcriptional profiles of neurons in the hippocampus influenced by stress that may underlie enhanced fear responses, single-nuclei RNAseq (snRNAseq) was used. snRNAseq was used because nuclei isolation overrepresents neurons compared to other cells (Supplementary Fig. 1). This is evident consistently across different labs, and publications [43, 49–51]. Overall, snRNAseq provided excellent resolution of neuronal subpopulations in the hippocampus.

**Fig. 4:**
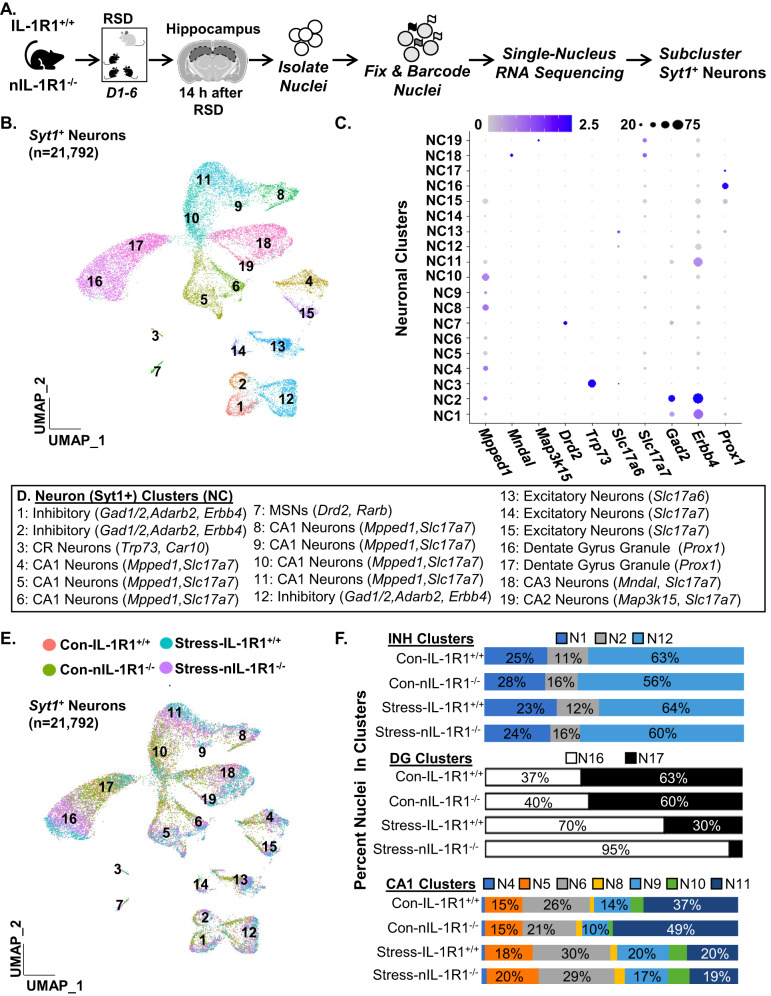
Stress and nIL-1R1 knockout influence single-nuclei RNAseq clustering and profiles. **A** Male IL-1R1^+/+^ and *Vglut2*^*+*^/IL-1R1^−/−^ (nIL-1R1^−/−^) mice were subjected to RSD or were undisturbed (control) and the hippocampus was dissected, pooled (3 mice per group) and nuclei were collected 14 h after RSD. Nucleus RNA profiles were determined by snRNA-seq. **B** UMAP clustering from a total of 21,792 neuronal nuclei identified 19 unique neuronal clusters. **C** Dot plot shows the expression of neuronal specific markers in the 19 neuronal clusters. UMAP plot with the distribution of cells based on the four treatments groups. **D** Annotation of each neuronal cluster based on markers found within the ConservedMarkers function. **E** Neuronal cluster distribution for each condition: Con-IL-1R1^+/+^, Con-nIL-1R1^−/−^, Stress-IL-1R1^+/+^, Stress-nIL-1R1^−/−^. **F** Percent nuclei represented in clusters of inhibitory neurons (INH), CA1 neurons, DG neurons for each experimental group: Con-IL-1R1^+/+^, Con-nIL-1R1^−/−^, Stress-IL-1R1^+/+^, Stress-nIL-1R1^−/−^. Clustering and differential expression were determined using uniform manifold approximation and projections (UMAP) clustering command in Seurat. Pooled samples for three replicates.

Here, IL-1R1^+/+^ and nIL-1R1^−/−^ mice were subjected to RSD and nuclei were isolated 14 h later (Fig. 4A). While different cell types were identified by snRNAseq (Supplementary Fig. 1), our focus was on neuronal profiles. Thus, neuronal nuclei (Syt1^+^) were subclustered for analysis. Figure 4B shows unsupervised UMAP clustering from 21,792 neurons (5000–6000 neurons/group). Nineteen clusters were identified with each condition represented. Figure 4C, D shows identities based on previous reports [39, 40] and ConservedMarkers (Seurat). Figure 4E highlights the distribution of clusters within each group. The DG clusters (NC16&17) had shifted distribution based on genotype and stress. Figure 4F shows the percentage of each cluster in INH, DG, and CA1 neurons for each group. Cluster distribution was determined via UMAP function (Seurat). Cluster distribution was influenced by stress for the DG and CA1 clusters (Fig. 4F). For instance, stress increased cluster percentage of DG N16 compared to controls (70% vs. 37%) and stress decreased CA1 N11 compared to controls (20% vs. 37%).

### Stress induced unique transcriptional patterns in hippocampal neurons that were dependent on neuronal IL-1R1 signaling

**Fig. 5:**
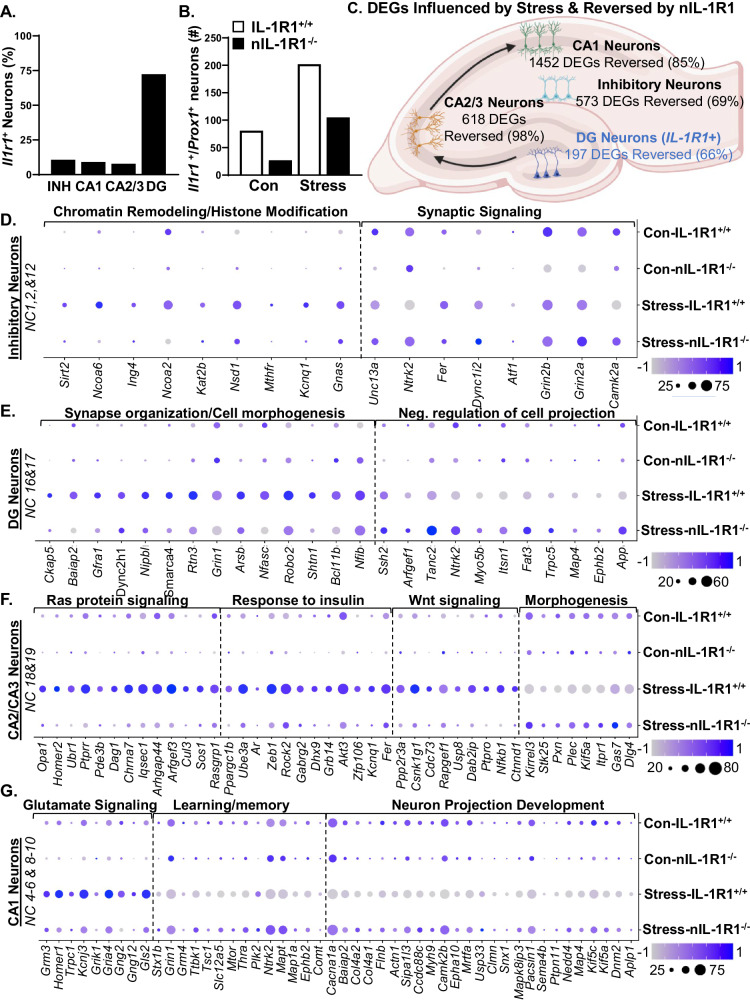
Stress-induced unique transcriptional patterns in hippocampal neurons that were dependent on neuronal IL-1R1 signaling. **A** The percentage of IL-1R1^+^ neurons in clusters of inhibitory (INH) neurons, CA1 neurons, DG neurons and CA2/3 neurons of the hippocampus for each condition: Con-IL-1R1^+/+^, Con-nIL-1R1^−/−^, Stress-IL-1R1^+/+^, Stress-nIL-1R1^−/−^. **B** Number of IL-1R1^+^/Prox1^+^ neurons from each condition. **C** Hippocampal diagram with the number of differentially expressed genes influenced by RSD and dependent on nIL-1R1 in DG, CA2/3, CA1 and INH neurons. Using Gene Ontology, DEGs (*p*adj < 0.05) were mapped to biological functions in inhibitory neurons, DG neurons, CA2/3 neurons, and CA1 neurons. **D** For inhibitory neurons, the following functions were used: chromatin remodeling (GO:0006338), histone modification (GO:0016570), and synaptic signaling (GO:0099536). **E** For CA1 neurons the following functions were used: glutamatergic synapse (GO:0098978), learning or memory (GO:0007611), and neuron projection development (GO:0031175). **F** For CA2/CA3 neurons the following functions were used: ras protein signaling (GO:0007265), response to insulin (GO:0032868), Wnt signaling (GO:0016055), and cell morphogenesis (GO:0000902). **G** For DG granule neurons the following functions were used: synapse organization (GO:0050808), cell morphogenesis (GO:0000902), and negative regulation of cell projection organization (GO:0031345).

Using the snRNAseq data, Fig. 5A shows counts of *Il1r1* in INH, CA1, CA2/3 and DG neurons in the hippocampus. In total, 75% of *Il1r1* counts were in *Prox1*^+^ granule neurons (Fig. 5B). Additionally, there were increased *Il1r1* counts in the DG after RSD compared to controls. These increases were attenuated in the Stress-nIL-1R1^−/−^ group. These data confirm IL-1R1 expression in DG neurons and reduction of IL-1R1 in the nIL-1R1^−/−^.

Next, differentially expressed genes (DEGs) were obtained from INH (NC1,2,&12), CA1 (NC4–6&9–10), CA2/3 (NC18&19) and DG neurons (NC 16&17) using FindMarkers (Seurat). Figure 5C depicts the DEGs influenced by RSD in INH neurons in which 69% (573 DEGs) were reversed in the Stress-nIL-1R1^−/−^ mice compared to Stress-IL-1R1^+/+^ mice. For CA1 neurons, 85% (1452 DEGs) of the RSD induced genes were reversed in the Stress-nIL-1R1^−/−^ mice. For CA2/CA3 neurons, 98% (618 DEGs) of the stress-induced genes were reversed in Stress-nIL-1R1^−/−^ mice. For DG neurons, 66% (197 DEGs) were reversed in the nIL-1R1^−/−^ mice (Fig. 5A). Thus, nIL-1R1 mediates neuronal transcriptome changes in each neuronal type of the hippocampus following RSD.

The DEGs reversed by nIL-1R1^−/−^ were used in GO (Metascape) [46]. In INH neurons, RSD influenced genes associated with chromatin remodeling, histone modification, and synaptic signaling (*p*adj. < 0.05). These increases were reversed in Stress-nIL-1R1^−/−^ mice (Fig. 5D). In DG neurons, stress influenced genes associated with synapse organization, cell morphogenesis, and negative regulation of cell projection, which all were reversed in Stress-nIL-1R1^−/−^ mice (Fig. 5E). In CA2/3 neurons, stress influenced genes associated with Ras signaling, insulin response, Wnt signaling, and cell morphogenesis (*p*adj. < 0.05, Fig. 5F). These stress influences were reversed in Stress-nIL-1R1^−/−^ mice (*p*adj. < 0.05, Fig. 5D). In CA1 neurons stress influenced genes associated with glutamatergic signaling, learning and memory, and neuron projection development (*p*adj. < 0.05). These increases were reversed in Stress-nIL-1R1^−/−^ mice (*p*adj. < 0.05, Fig. 5G). Thus, increased neuronal IL-1 signaling after RSD influences distinct pathways (direct/indirect) in specific neurons of the hippocampus.

### Stress induced unique canonical pathways, regulators, and cell-to-cell communication in hippocampal neurons dependent on nIL-1R1 signaling

**Fig. 6:**
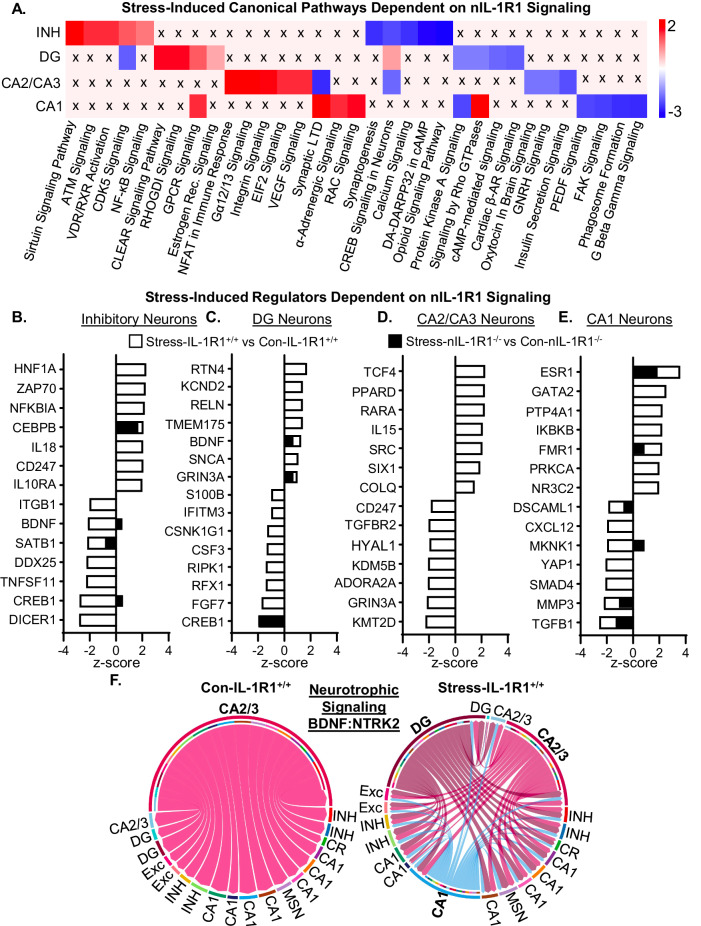
Stress uniquely altered neuronal canonical pathways, regulators, and communications pathway via neuronal IL-1 signaling. **A** Heatmap shows IPA canonical pathways influenced by RSD and reversed by nIL-1R1^−/−^ from DEGs (*p*adj < 0.05) from each subtype of neuron in the hippocampus. **B**–**E** Top regulators from IPA altered by stress within IL-1R1^+/+^ and nIL-1R1^−/−^ groups. The IL-1R1 comparison is denoted with white bars and the nIL-1R1^−/−^ comparison is denote with black bars. **F** CellChat chord diagram demonstrating neurotrophic signaling in Control-IL-1R1^+/+^ mice and Stress-IL-1R1^+/+^. Neurotrophic signaling was not significantly overexpressed in either nIL-1R1 groups.

Next, DEGs that were influenced by stress and reversed in Stress-nIL-1R1^−/−^ mice were used in IPA. Pathways with the highest (+) and lowest (−) *z*-score were selected in each neuronal subtype (Fig. 6A). Several inflammatory-related pathways were increased in different classes of neurons, including NF-κB, NFAT, GPCR, and integrin signaling. These increases were prevented by nIL-1R1^−/−^. These data are consistent with a previously published using bulk-RNAseq [47]. Notably, INH and CA1 neurons had reduced pathways of neuronal activation or synaptic strength (CREB signaling, calcium signaling, and protein kinase A signaling). DG neurons had increased CREB signaling and was prevented in nIL-1R1^−/−^ mice (Fig. 6A). This is consistent with hippocampal pCREB labeling (Fig. 3H).

Next, regulators with the highest (+) and lowest (−) *z*-score were selected. Again, stress induced several inflammatory pathways that were nIL-1R1 dependent (Fig. 6B–E). In the INH neurons, several regulators of inflammation were increased by stress and dependent on nIL-1R1 signaling (NFKBIA, IL18, and IL10RA). There was a decrease in regulators of neuronal function/activation (brain-derived neurotrophic factor (BDNF) and CREB1) and DNA modification (SATB1 and DICER1). In the DG neurons, several regulators related to fear memory were increased by stress and were dependent on nIL-1R1, including KCND2 and BDNF [52, 53]. There was reduced CREB1 in the Stress-nIL-1R1^−/−^, consistent with pCREB labeling (Fig. 3H) and IPA pathways (Fig. 6A). In the CA2/CA3 neurons, several regulators of inflammation were increased by stress and were dependent on nIL-1R1 signaling, including PPARD and IL15. Meanwhile, regulators of neuronal activation (ADORA2A and GRIN3A) and DNA modification (KDM5B and KMT2D) were reduced. In CA2/CA3 neurons, several regulators of inflammation were increased by stress and were dependent on nIL-1R1 signaling (IKBKB and NR3C2). Meanwhile, RSD decreased regulators of synaptic complexity (YAP1 and MMP3) and TGF-β pathways (SMAD4 and TGFB1). Collectively, stress-induced alterations to neuronal subpopulations regulating pathways related to neuronal activation and inflammation.

Using cellchat, overexpressed pathways were obtained. Compared to Con-IL-1R1^+/+^, Stress-IL-1R1^+/+^ mice had increased neurotrophic signaling from the DG and CA1 (each color denotes a difference source). This signaling pathway was not enhanced in the nIL-1R1 groups (not shown). This is consistent with the stress-induced increase BDNF a regulator above, which was attenuated with the nIL-1R1^−/−^ (Fig. 6F). Overall, stress induced myriad changes in hippocampal neurons that were mediated by neuronal IL-1 signaling.

## Discussion

RSD causes sensitization in several cellular compartments including neurons [5]. Our previous studies indicate that neuronal *Vglut2*^+^/IL-1R1 was critical for stress-sensitization. Additionally, stress prolonged fear extinction, increased freezing during extinction recall, and increased IL-1β expression in the hippocampus following fear conditioning [20]. Novel aspects of this study were that stress-enhanced contextual fear memory persisted 29 days later. Moreover, the stress-enhanced fear memory and hippocampal pCREB activation were neuronal IL-1R1-dependent, but microglia/monocyte independent. Last, snRNAseq shows myriad stress-dependent influences on neuronal subpopulations in the hippocampus dependent on nIL-1R1. Overall, we provide new data that IL-1R1-mediated signaling (monocytes/microglia independent) in glutamatergic neurons after RSD enhanced neuronal reactivity and contextual fear memory. We provide a novel neuronal RNA signature in the hippocampus (single-nuclei level) that represents the IL-1R1-dependent influence of RSD. These RNA profiles in neurons, especially DG neurons, may represent the molecular basis of neuronal stress-sensitization and has relevance toward understanding the neurobiology of PTSD.

One interesting aspect of this study was that stress-induced enhancement of fear memory and neuronal reactivity with increased pCREB activation were independent of monocytes/microglia. Consistent with previous work, PLX5622 depleted microglia and reduced accumulation of CD45^+^ cells (monocytes) in the hippocampus after RSD [5, 54, 55]. Microglia depletion prior to RSD, however, had no effect on the stress-induced increase in contextual fear memory. Previous studies report that microglia depletion increased freezing during fear conditioning [56, 57]. For instance, using PLX3397, mice had increased freezing 35 days following fear acquisition. The authors interpreted these data to indicate that microglia mediated fear extinction or “forgetting” [56]. Thus, there may be memory effects of microglia depletion with PLX3397, which is a CSFR1 antagonist and c-kit inhibitor [58]. Nonetheless, microglia depletion with PLX5622 did not influence the response to stress and fear memory was still enhanced. Microglia depletion also did not reverse the stress induction in neuronal reactivity. Collectively we interpret these data to indicate that the stress-induced fear memory was independent of monocytes/microglia.

A key finding was that stress-induced enhancement of fear memory and neuronal reactivity with pCREB activation were dependent on neuronal IL-1R1. The involvement of IL-1R1 in stress-induced fear response is consistent with other studies reporting that IL-1β and IL-1R1 are associated with fear memory [59–63]. For instance, stress-enhanced fear learning (SEFL) increased IL-1β expression in the DG and treatment with IL-1RA prevented SEFL [59]. In a study of amyloid-β accumulation, global IL-1R1^−/−^ reversed fear memory deficits. The novelty here is that we show these responses of IL-1R1 are specifically in *Vglut2*^+^ neurons, which are highly expressed in the DG of the hippocampus [23, 30]. There is evidence that *Vglut2* is expressed on other regions at baseline [30]. Notably, nIL-1R1^−/−^ did not affect microglia activation, monocyte recruitment, and IL-1β after RSD. This is consistent with previous RSD studies [29, 47]. Additionally, pCREB activation (i.e., neuronal reactivity) in the DG after stress and fear conditioning was independent of microglia/monocytes but was dependent on nIL-1R1. The use of pCREB to determine neuronal re-activity is based on our previous studies [7, 29]. Other studies have validated that pCREB is predominantly in the granule cell layer of the DG [48, 64–66]. While pCREB expression was not assessed 28 days following fear conditioning, our previous studies show pCREB expression was only increased in stress-sensitized mice that received an acute stress at 24 days [7]. These data are consistent with the pCREB in the hippocampus of mice exposed to RSD and then fear conditioning at D9. We interpret these data as increased pCREB in mature neurons, but it is plausible that pCREB in the subventricular zone is increased in newly formed neurons [64]. Previously, we did not detect an increase in neurogenesis in the hippocampus 14 h after RSD [67], but those mice were not exposed to fear conditioning. Thus, further studies are needed to confirm maturity of the pCREB^+^ neurons. Overall, these interpretations align with the snRNAseq data showing that CREB Signaling in DG neurons was increased after stress and was dependent on nIL-1R1 (Fig. 6A). In addition, the dorsal DG is critical for both the encoding and retrieving of fear memories. For example, optogenetic inhibition of the dorsal DG decreased freezing during acquisition [68]. Here, stress-enhanced contextual fear and neuronal reactivity was dependent on nIL-1R1. While nIL-1R1^ko^ prevented enhanced fear memory at 7 days after RSD, it is unclear if nIL-1R1^ko^ would also prevent enhanced pCREB reactivity and fear memory at 28 days. Based on our previous studies with nIL-1R1^ko^ and stress-sensitization, we believe that neuronal IL-1R1 will have a key role. Nonetheless, we acknowledge there are other pathways that promote long-term sensitization in the context of fear conditioning, independent of neuronal IL-1R1. Collectively, we interpret these data to indicate that there was long-lasting sensitization of IL-1R1^+^ hippocampal neurons after RSD.

Another relevant finding was that stress-induced *IL-1β* RNA expression in the DG/hippocampus was independent of microglia/monocytes. While stress robustly increased *IL-1β* RNA in the hippocampus (RT-qPCR/RNAscope), it was unaffected by microglial depletion. Hippocampal *IL-1β* RNA was also unaffected by nIL-1R1^−/−^. Microglia and monocytes both express IL-1β after RSD [13, 67, 69] and communicate with endothelia to increase prostaglandins [14]. This pathway mediated anxiety in the open-field [14]. In coronal brain sections, RSD-induced IL-1β RNA was reduced by microglia depletion with PLX5622 [67]. This coronal section included several brain regions and was not specific to the DG/hippocampus [55]. Here, microglia depletion did not prevent IL-1β RNA expression in the DG/hippocampus after RSD. The interpretation is that non-myeloid cells may express IL-1β in the hippocampus. Based on location of the RNAscope labeling, neurons are likely expressing IL-1β. Thus, hippocampal IL-1R1 signaling is associated with neuronal sensitization, but the IL-1β in this region is not primarily from monocytes/microglia.

Relevant to neuronal sensitization after RSD, novel snRNAseq data revealed myriad stress-dependent influences on neuronal subpopulations (INH, CA1, CA2/3, and DG) that were dependent on nIL-1R1. As expected, DG neurons contained the highest percentage of *Il1r1* and was decreased in the nIL-1R1^−/−^ mice after RSD. These data support previous studies showing that IL-1R1 is highly expressed in the DG compared to other CNS regions [23, 29, 30] and that the Vglut2-Cre targets the hippocampus (Liu et al. [21]). Using GO, stress-influenced pathways associated with synaptic signaling (INH), glutamatergic signaling (CA1), learning and memory (CA1), synapse organization/cell morphogenesis (DG), and negative regulation of cell projection (DG). All these genes/pathways induced by stress were nIL-1R1 dependent. There were increases in pathways associated with neuroinflammation (NF-κB, T-Cell receptor signaling, CLEAR signaling) and were nIL-1R1 dependent. These data align with previous results of bulk-RNAseq of the hippocampus after RSD [47]. Also, INH transcriptome changes point to a disruption in the excitatory/inhibitory balance, which may alter spatial memory and fear behaviors [70–72]. Collectively, stress influenced the transcription of multiple neuronal subtypes in the hippocampus relating to synaptic plasticity and were nIL-1R1 dependent.

Another relevant finding was the influence of stress on the DG neurons. These are IL-1R1-expressing neurons. There was increased CREB signaling, estrogen-receptor (ER) signaling, synapse organization, cell morphogenesis, and calcium signaling after RSD. These stress-induced pathways were nIL-1R1-dependent. Data above and previous work has implicated each of these DG pathways in increased fear memory and synaptic plasticity [73]. A previous study showed ER signaling mediates hippocampal-based memory by interacting with metabotropic glutamate receptors and inducing CREB signaling in neurons [74]. When neuronal-derived 17β-estradiol was depleted, contextual fear extinction was decreased [75]. Thus, ER pathways are involved in contextual memory via glutamatergic and CREB signaling. There was also a decrease in Cdk5 signaling in the DG neurons after RSD (nIL-1R1-dependent). Cdk5 is associated with morphogenesis, synapse formation, and contextual memory [76]. Moreover, there was significant inhibition of stress responses in low IL-1R1-expressing neurons with nIL-1R1^−/−^ including INH, CA1, and CA2/3 neurons. As noted, IL-1R1 is highly expressed on granule cells of the DG. These DG neurons receive input from many sources and relay signals to the CA2/3 and CA1 [77]. Thus, preventing IL-1R1 signaling in DG neurons had profound downstream effects on the CA2/CA3, CA1, and INH neurons. Collectively, these IL-1R1 dependent pathways are critical in the sensitization of DG neurons after RSD.

snRNAseq revealed that stress uniquely altered pathways associated with increased glutamate signaling, synaptic plasticity, and long-term potentiation/depression (LTP/LTD) in the CA1 neurons. The influence of stress on CA1 neurons is likely downstream to the sensitization of the DG neurons. CA1 neurons increased glutamate signaling, synaptic LTD, and adrenergic signaling. Additionally, there was stress-induced decreases in FAK and PKA signaling, which are associated with neuronal transmission and neurotransmitter release [78, 79]. GO showed changes in the CA1 associated with learning and memory, and neuronal projection development. This aligns with previous data demonstrating that nIL-1R1^−/−^ prevented y-maze deficits after RSD [47], dependent on CA1 activity [80, 81]. Also, BDNF signaling was increased in DG neurons, and it was augmented in cell-to-cell signaling via CellChat. These effects of stress were absent in the Stress-nIL-1R1^−/−^ mice. These data are consistent with studies showing that fear conditioning acquisition and recall were associated with increased BDNF in the hippocampus [82]. Specifically, increased hippocampal BDNF was associated with increased contextual fear [52, 83]. Other reports using restraint and chronic mild stress, showed decreased hippocampal BDNF. Nonetheless, these reports only assessed global *Bdnf* mRNA or protein in the hippocampus [84, 85]. Here, neuron subpopulations were evaluated after RSD and BDNF signaling was increased in DG neurons and decreased in INH of the hippocampus. Thus, BDNF and related genes were selectively affected by stress in hippocampal neurons. Furthermore, we provide evidence that increased BDNF-associated pathways in the DG were nIL-1R1 dependent. Thus, stress enhanced neurotrophic signaling in the DG and could be a key component of neuronal sensitization and enhanced fear memory after RSD.

In conclusion, neuronal IL-1R1 signaling has a critical role in mediating enhanced fear memory after RSD. Here, neuronal IL-1R1 knockout prevented stress-induced fear memory and neuronal activation in the hippocampus. Additionally, neuronal transcriptome changes across the hippocampus after RSD were dependent on neuronal IL-1R1 signaling. Although fear conditioning involves a complex network, we identified IL-1R1 signaling in *Vglut2*^+^ neurons as basis of sensitization of DG neurons and the mechanism by which there is enhanced fear memory after RSD.

## Supplementary information

Supplement Methods

Supplemental Figure

Supplemental Figure Legend

## Data Availability

The single-nuclei data discussed have been deposited in NCBI’s Gene Expression Omnibus and are accessible through GEO Series accession number GSE253687.

## Code availability

Code is available upon request.
