# Supplementary material for: Enhanced fear memory after social defeat in mice is dependent on interleukin-1 receptor signaling in glutamatergic neurons
Source: Mol Psychiatry. 2024 Mar 8;29(8):2321–34. doi: 10.1038/s41380-024-02456-1 (PMC11412902; doi:10.1038/s41380-024-02456-1)
Supplement: Supplementary file 1 — Supplement Methods [file 41380_2024_2456_MOESM1_ESM.doc]

**Supplement Methods**

*Mice:* Male wild type C57BL/6 (5-7 weeks) and CD-1 aggressor mice were purchased from Charles River Laboratories (Wilmington, MA). Transgenic IL-1R1^+/+^ (Cre^-^) and neuronal (*Vglut2^+^*)/IL-1R1^-/-^ (Cre^+^) mice were bred in-house ^1-3^. IL-1R1^+/+^ (Cre^-^) and neuronal (*Vglut2^+^*)/IL-1R1^+/+^ (Cre^+^) were co-housed during repeated social defeat. Treatment groups and behavior order were randomized. Mice had ad libitum access to food and water and were housed under a 12 h light/dark cycle. For these current experiments, only male mice were used. All procedures were in accordance with the NIH Guidelines and were approved by the Ohio State University Institutional Laboratory Animal Care and Use Committee.

*Repeated Social Defeat****:*** Mice were subjected to Repeated Social Defeat (RSD) as previously described ^4, 5^. In brief, a male CD-1 aggressor mouse was placed into the home cage of experimental mice (3 mice per cage) for two hours (between 16:00 to 18:00) per night for six consecutive nights. During the two hours, submissive behaviors (e.g., upright posture, fleeing, crouching) were observed to ensure experimental mice showed signs of defeat. A new CD-1 aggressor was introduced to the cage if no attack occurred within 3-5 minutes or if an experimental mouse defeated the CD-1 aggressor. At the end of the 2 h period, aggressor mice were removed, and experimental mice were left undisturbed in their home cages until the following day when the paradigm was repeated. To avoid habituation, different aggressors were used on consecutive nights. The health of the experimental mice was monitored carefully throughout the experiments. Experimental mice that were significantly wounded, injured, or moribund were removed from the study. Similar to previous studies, less than 5% of mice met the early removal criteria ^6^. Control mice were left undisturbed in a separate room and in their home cages. Only male mice were used in this study. Previous studies have established RSD in females, using modified social defeat with male DREADD aggressors^7, 8^. Using this model, female mice have neuronal activation and microglia reactivity in stress responsive regions, monocyte trafficking to the brain, and anxiety-like behavior after social defeat^9^.

*Plexxikon (PLX) 5622 Administration:* PLX5622 was provided by Plexxikon Inc. (Berkley, CA) and formulated in AIN-76A rodent chow by Research Diets at a concentration of 1,200 mg/kg. Standard AIN-76A diet was provided as a vehicle control. Mice were provided ad libitum access to PLX5622 or vehicle diet for 7 days to deplete microglia prior to RSD. This dose and time was validated and results in ~96% depletion of microglia from the brain ^10^.

*Fear Conditioning****:*** Fear conditioning was conducted as described with some modifications ^11^. In brief, the fear conditioning paradigm was initiated on either d7 or d27 (1 day or 22d after the cessation of social defeat). For the acquisition trial, mice were habituated in a clear (16inx16in) plexiglass container with a shock grid and gray wall inserts for 90s followed by a 30s (2000 Hz, 70db) tone where a 0.5 mA shock co-terminated during the last two seconds. The tone/shock pairing repeated five times with a 30s inter-trial interval (ITI). Mice were returned to their home cage after the last tone/shock pairing for 1 day. For the contextual trial, mice were habituated in the behavioral suite for 45 minutes then placed in the plexiglass box (context A) and time spent freezing was recorded for 10 minutes. Mice were returned to home cages for 1 day. For the cued trial, mice were habituated for 90s in a new context (context B) with checkered wall and floor inserts and a banana extract scent. Next, a tone played five time with 30 second ITIs. Experimenters were blinded to the group conditions and data were analyzed (e.g., time spent Percent freezing) using Fusion software (Omnitech Electronics). Each cohort was randomized, and experimenters were blinded to the treatments and data were analyzed using Fusion software.

*pCREB, Iba1 and CD45 detection:* Immunohistochemical analyses were completed as described ^6^. In brief, the brain was post fixed in 4% formaldehyde and then cryoprotected with 30% sucrose. The brain was sectioned (30 um) and sections were washed (PBS with 0.1% Triton X-100), blocked (5% normal donkey serum and 0.3% Triton X-100), and then incubated with primary antibodies. Hippocampal sections were incubated with rat anti-mouse CD45 (1:500, Bio-Rad; #MCA1388), rabbit anti-mouse Iba1 (1:1000, Wako; #019-19741), or rat anti-mouse pCREB (1:1000, Cell Signaling; #9198) antibodies overnight at 4°C. Next hippocampal sections were washed and incubated with fluorochrome-conjugated secondary antibodies: Donkey anti-rabbit 647 or Donkey anti-goat 488 (Thermo Fisher Scientific). Labeled sections were washed, mounted on slides and imaged using an EVOS M7000 imaging system (10x or 20x magnification). Images were analyzed using ImageJ to determine cell counts, percent area, or mean fluorescent intensity of the fluorescent labeling. Experimenters were blinded to the treatments during image capture and analysis.

*RNA isolation and qPCR****.*** Total RNA was extracted from the hippocampus using Tri-Reagent (Sigma-Aldrich, #T9424) and cDNA was generated from the High-Capacity Reverse Transcription Kit (Applied Biosystems, #4374966). Quantitative real-time (q)-PCR for IL-1β expression (mm00434228_m1) was completed using a TaqMan Gene Expression assay in which cDNA was amplified using qPCR where the target gene(s) and reference gene (GAPDH) was amplified concurrently using an oligonucleotide probe with a 5’ fluorescent reporter dye (FAM) and 3’ quencher (NFQ). Fluorescence was determined using QuantStudio 3 Real-Time PCR System (Applied Biosystem). Data was generated using the comparative threshold cycle (ΔΔCT) method and date were expressed as fold change compared to controls.

*In situ hybridization of IL-1β:* RNAscope was performed as previously described ^6^ to detect Il-1b in the hippocampus using a Multiplex Fluorescent Reagent Kit v2 (Advanced Cell Diagnostics; #323100). In brief, tissue sections were washed, heated to 60°C for 30 minutes, post-fixed, and dehydrated in ethanol. Sections were treated with hydrogen peroxide for 10 minutes and co-detection antigen retrieval was performed for 10 minutes. The following day, sections were treated with Protease III for 30 minutes and the probe (IL-1β-C1; #316891) was hybridized for 2 h at 40°C. Probe Amplification performed according to manufacturer’s instructions and signal was developed using an Opal 690 dye (Akoya Biosciences; #FPI1497001KT) and counterstained with DAPI. Images were captured using a Leica SP8 confocal imaging system at 63X magnification percent area *IL-1β* RNA was quantified using ImageJ**.** Experimenters were blinded to the treatments during image capture and analysis.

*Nuclei isolation:* Hippocampi (n=3) were extracted and pooled. Pooled samples were homogenized using dounce homogenizers and a homogenization buffer (1.5 M NIM1 Buffer with 250mM sucrose, 25mM KCl, 5mM MgCl2, 10mM Tris Buffer pH 8, 1uM DTT, 0.4U/uL Enzymatics RNAase-Inhibitor (#Y9240L), 0.2U/uL Superase-Inhibitor (Thermo Fisher Scientific, #AM2694), and .1% Triton X-100). Hippocampi homogenates were filtered (40uM strainer) and clarified. Resulting homogenates were resuspended in a PBS buffer with RNase Inhibitors (0.05U/ul of Enzymatics RNAase-Inhibitor and Superase-Inhibitor) and re-pelleted. To remove myelin debris, homogenates were incubated with Myelin Removal Beads II (Miltenyi Biotec, #130-096-731) for 15 minutes at 4°C. Homogenates were washed (50% PBS and 50% PBS + 1% BSA) and re-pelleted. Supernatant was removed and samples were resuspended in wash buffer. One LS column (Miltenyi Biotec, # 130-042-401) per sample was used to filter the samples followed by pelleting and resuspending in wash buffer. Nuclei were counted with AO/PI (Logos Biosystems, #F23001) on a Luna-FL Cell Counter and fixed with a Nuclei Fixation Kit (Parse Biosciences, #SB1003) per the manufacturer’s instructions followed by freezing at -80°C in a Mr. Frosty (Thermo Fisher Scientific, #5100-001).

*Single nuclei barcoding and sub-library generation:* The Parse Biosciences Whole Transcription Kit was used to barcode and generate eight separate sub-libraries with 12,500 nuclei per sub-library according to the manufacturer’s instructions. DNA concentration was measured by Qubit 4 Fluorometer and a Qubit dsDNA HS Assay Kit (Thermo Fisher Scientific, #Q32851). A Bioanalyzer 2100 with a High Sensitivity DNA Assay chip was used to control quality of sub-libraries before samples were sequenced. RNA was sequenced at a depth of 40,000 reads per nuclei using a NovaSeq S4 at the Advanced Genomics Core at the University of Michigan Advanced Genomics Core.

*SnRNAseq Data Processing:* Each fastq.gz file was downloaded and aligned to the Genome Reference Consortium Mouse Reference 39 (mm39) using the Parse Biosciences pipeline. Matrices were downloaded and filtered in RStudio using Seurat (v4.1.1)^12^. Nuclei showing greater than 20% mitochondrial DNA were filtered out prior to clustering. The *Syt1*+ were subset to analyze neurons. After clustering using Uniform Manifold Approximation and Projection (UMAP) cell identification was performed with established markers: neurons (*Syt1*), CA1 neurons (*Mpped1*), CA2/3 (*Mndal*,*Map3k15*), DG neurons (*Prox1*), inhibitory neurons (*Gad1&2, Adarb2, Erbb4*), excitatory neurons (*Slc17a7*), Cajal–Retzius cells (*Trp73*, *Car10*), and medium spiny neurons (*Drd2*, *Rarb*)^13-18^. Differential gene expression from p-adjusted values was performed using the FindMarkers feature of Seurat with Model-based Analysis of Single-cell Transcriptomics (MAST)^19^. Pathway and master regulators analysis was performed with Ingenuity Pathway Analysis (IPA; Qiagen)^20^.

Code availability: Code is available upon request. The single nuclei data discussed have been deposited in NCBI’s Gene Expression Omnibus and are accessible through GEO Series accession number GSE253687.

*Statistical analysis:* Data was analyzed GraphPad Prism 9 with T-tests or two-way ANOVAs to determine main effects and interactions between factors. Tukey HSD was used for *post-hoc* analysis when main effects or interactions were significant. Grubbs outlier test was used to determine outliers (Q=1%). Samples that qualified as outliers were removed from all analyses. All figures are means with s.e.m. Individual data points >2 standard deviations above and below the mean were considered outliers. Samples sizes were determined based on previously published power analyses and studies using the same experimental approaches^1, 6, 21^. All experiments pass the Shapiro-Wilk test of normality.

Sample Sizes

| Fig.1-Fear Conditioning (D7)  1 Cohort | Control (n=5) | Stress (n=5) | No Shock Control (n=5) | No Shock Stress (n=5) |
| --- | --- | --- | --- | --- |
| Fig.1-Fear Conditioning (D28)  1 Cohort | Control (n=6) | Stress (n=9) | No Shock Control (n=5) | No Shock Stress (n=5) |
| Fig.2-Fear Conditioning  5 Cohorts | Con-Veh (n=15) | Con-PLX (n=15) | Stress-Veh (n=15) | Stress-PLX (n=15) |
| Fig.2-pCREB | Con-Veh (n=3) | Con-PLX (n=3) | Stress-Veh (n=4) | Stress-PLX (n=5) |
| Fig.2-IBA1 | Con-Veh (n=6) | Con-PLX (n=6) | Stress-Veh (n=6) | Stress-PLX (n=6) |
| Fig.2-CD45 | Con-Veh (n=4) | Con-PLX (n=5) | Stress-Veh (n=6) | Stress-PLX (n=6) |
| Fig.2-RNAscope | Con-Veh (n=3) | Con-PLX (n=4) | Stress-Veh (n=4) | Stress-PLX (n=4) |
| Fig.3-Fear Conditioning  3 cohorts | Con-IL-1R1^+/+^ (n=13) | Con-nIL-1R1^-/-^(n=9) | Stress- IL-1R1^+/+^ (n=15) | Stress-nIL-1R1^-/-^ (n=11) |
| Fig.3-pCreb | Con-IL-1R1^+/+^ (n=4) | Con-nIL-1R1^-/-^(n=6) | Stress- IL-1R1^+/+^ (n=4) | Stress-nIL-1R1^-/-^ (n=4) |
| Fig.3-IBA1 | Con-IL-1R1^+/+^ (n=3) | Con-nIL-1R1^-/-^(n=5) | Stress- IL-1R1^+/+^ (n=4) | Stress-nIL-1R1^-/-^ (n=8) |
| Fig.3-CD45 | Con-IL-1R1^+/+^ (n=4) | Con-nIL-1R1^-/-^(n=6) | Stress- IL-1R1^+/+^ (n=6) | Stress-nIL-1R1^-/-^ (n=8) |
| Single nuclei RNA-seq (pooled) | Con-IL-1R1^+/+^ (n=3) | Con-nIL-1R1^-/-^(n=3) | Stress- IL-1R1^+/+^ (n=3) | Stress-nIL-1R1^-/-^ (n=3) |

# References

1. DiSabato DJ, Yin W, Biltz RG, Gallagher NR, Oliver B, Nemeth DP *et al.* IL-1 Receptor-1 on Vglut2+ neurons in the hippocampus is critical for neuronal and behavioral sensitization after repeated social stress. *Brain, Behavior, & Immunity-Health* 2022; **26:** 100547.

2. Liu X, Nemeth DP, McKim DB, Zhu L, DiSabato DJ, Berdysz O *et al.* Cell-Type-Specific Interleukin 1 Receptor 1 Signaling in the Brain Regulates Distinct Neuroimmune Activities. *Immunity* 2019; **50**(2)**:** 317-333 e316.

3. Liu X, Yamashita T, Chen Q, Belevych N, McKim DB, Tarr AJ *et al.* Interleukin 1 type 1 receptor restore: a genetic mouse model for studying interleukin 1 receptor-mediated effects in specific cell types. *J Neurosci* 2015; **35**(7)**:** 2860-2870.

4. Lisboa SF, Niraula A, Resstel LB, Guimaraes FS, Godbout JP, Sheridan JF. Repeated social defeat-induced neuroinflammation, anxiety-like behavior and resistance to fear extinction were attenuated by the cannabinoid receptor agonist WIN55,212-2. *Neuropsychopharmacology : official publication of the American College of Neuropsychopharmacology* 2018; **43**(9)**:** 1924-1933.

5. Weber MD, McKim DB, Niraula A, Witcher KG, Yin W, Sobol CG *et al.* The Influence of Microglial Elimination and Repopulation on Stress Sensitization Induced by Repeated Social Defeat. *Biological psychiatry* 2019; **85**(8)**:** 667-678.

6. Yin W, Swanson SP, Biltz RG, Goodman EJ, Gallagher NR, Sheridan JF *et al.* Unique brain endothelial profiles activated by social stress promote cell adhesion, prostaglandin E2 signaling, hypothalamic–pituitary–adrenal axis modulation, and anxiety. *Neuropsychopharmacology : official publication of the American College of Neuropsychopharmacology* 2022; **47**(13)**:** 2271-2282.

7. Dion-Albert L, Cadoret A, Doney E, Kaufmann FN, Dudek KA, Daigle B *et al.* Vascular and blood-brain barrier-related changes underlie stress responses and resilience in female mice and depression in human tissue. *Nature communications* 2022; **13**(1)**:** 164.

8. Takahashi A, Chung JR, Zhang S, Zhang H, Grossman Y, Aleyasin H *et al.* Establishment of a repeated social defeat stress model in female mice. *Scientific reports* 2017; **7**(1)**:** 12838.

9. Yin W, Gallagher NR, Sawicki CM, McKim DB, Godbout JP, Sheridan JF. Repeated social defeat in female mice induces anxiety-like behavior associated with enhanced myelopoiesis and increased monocyte accumulation in the brain. *Brain, behavior, and immunity* 2019; **78:** 131-142.

10. Witcher KG, Bray CE, Chunchai T, Zhao F, O'Neil SM, Gordillo AJ *et al.* Traumatic Brain Injury Causes Chronic Cortical Inflammation and Neuronal Dysfunction Mediated by Microglia. *The Journal of Neuroscience* 2021; **41**(7)**:** 1597-1616.

11. Tipps ME, Raybuck JD, Buck KJ, Lattal KM. Delay and trace fear conditioning in C57BL/6 and DBA/2 mice: issues of measurement and performance. *Learning & memory* 2014; **21**(8)**:** 380-393 %@ 1072-0502.

12. Hao Y, Hao S, Andersen-Nissen E, Mauck WM, 3rd, Zheng S, Butler A *et al.* Integrated analysis of multimodal single-cell data. *Cell* 2021; **184**(13)**:** 3573-3587 e3529.

13. O'Neil SM, Hans EE, Jiang S, Wangler LM, Godbout JP. Astrocyte immunosenescence and deficits in interleukin 10 signaling in the aged brain disrupt the regulation of microglia following innate immune activation. *Glia* 2022; **70**(5)**:** 913-934.

14. Zhong S, Wang M, Zhan Y, Zhang J, Yang X, Fu S *et al.* Single-nucleus RNA sequencing reveals transcriptional changes of hippocampal neurons in APP23 mouse model of Alzheimer’s disease. *Bioscience, Biotechnology, and Biochemistry* 2020; **84**(5)**:** 919-926.

15. Kalinina A, Lagace D. Single-Cell and Single-Nucleus RNAseq Analysis of Adult Neurogenesis. *Cells* 2022; **11**(10).

16. Franjic D, Skarica M, Ma S, Arellano JI, Tebbenkamp ATN, Choi J *et al.* Transcriptomic taxonomy and neurogenic trajectories of adult human, macaque, and pig hippocampal and entorhinal cells. *Neuron* 2022; **110**(3)**:** 452-469 e414.

17. Witcher KG, Bray CE, Chunchai T, Zhao F, O'Neil SM, Gordillo AJ *et al.* Traumatic brain injury causes chronic cortical inflammation and neuronal dysfunction mediated by microglia. *Journal of Neuroscience* 2021; **41**(7)**:** 1597-1616.

18. Rosenberg AB, Roco CM, Muscat RA, Kuchina A, Sample P, Yao Z *et al.* Single-cell profiling of the developing mouse brain and spinal cord with split-pool barcoding. *Science* 2018; **360**(6385)**:** 176-182.

19. Finak G, McDavid A, Yajima M, Deng J, Gersuk V, Shalek AK *et al.* MAST: a flexible statistical framework for assessing transcriptional changes and characterizing heterogeneity in single-cell RNA sequencing data. *Genome biology* 2015; **16**(1)**:** 1-13.

20. Kramer A, Green J, Pollard J, Jr., Tugendreich S. Causal analysis approaches in Ingenuity Pathway Analysis. *Bioinformatics* 2014; **30**(4)**:** 523-530.

21. DiSabato DJ, Nemeth DP, Liu X, Witcher KG, O’Neil SM, Oliver B *et al.* Interleukin-1 receptor on hippocampal neurons drives social withdrawal and cognitive deficits after chronic social stress. *Molecular psychiatry* 2021; **26**(9)**:** 4770-4782.
