# Supplementary material for: Enhanced fear memory after social defeat in mice is dependent on interleukin-1 receptor signaling in glutamatergic neurons
Source: Mol Psychiatry. 2024 Mar 8;29(8):2321–34. doi: 10.1038/s41380-024-02456-1 (PMC11412902; doi:10.1038/s41380-024-02456-1)
Supplement: Supplementary file 2 — Supplemental Figure [file 41380_2024_2456_MOESM2_ESM.pdf]

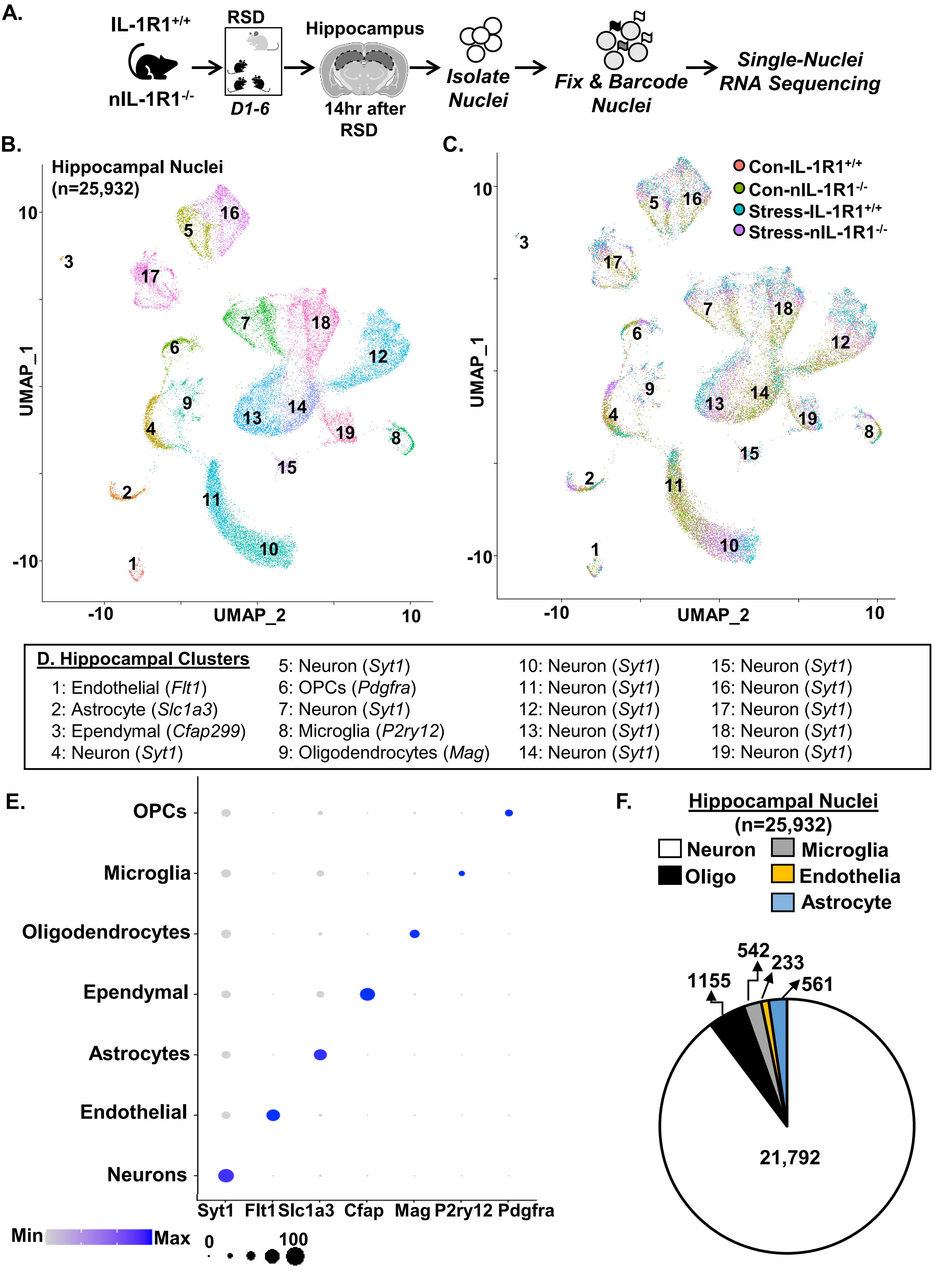
A.
IL-1R1+/+
nIL-1R1-/-
RSD
D1-6
Hippocampus
14hr after RSD
Isolate Nuclei
Fix & Barcode Nuclei
Single-Nuclei RNA Sequencing
B.
Hippocampal Nuclei (n=25,932)
UMAP_1
UMAP_2
10
-10
C.
Con-IL-1R1+/+
Con-nIL-1R1-/-
Stress-IL-1R1+/+
Stress-nIL-1R1-/-
D. Hippocampal Clusters
1: Endothelial (Flt1)
2: Astrocyte (Slc1a3)
3: Ependymal (Cfap299)
4: Neuron (Syt1)
5: Neuron (Syt1)
6: OPCs (Pdgfra)
7: Neuron (Syt1)
8: Microglia (P2ry12)
9: Oligodendrocytes (Mag)
10: Neuron (Syt1)
11: Neuron (Syt1)
12: Neuron (Syt1)
13: Neuron (Syt1)
14: Neuron (Syt1)
15: Neuron (Syt1)
16: Neuron (Syt1)
17: Neuron (Syt1)
18: Neuron (Syt1)
19: Neuron (Syt1)
E.
OPCs
Microglia
Oligodendrocytes
Ependymal
Astrocytes
Endothelial
Neurons
Syt1 Flt1 Slc1a3 Cfap Mag P2ry12 Pdgfra
Min Max
0 100
F.
Hippocampal Nuclei (n=25,932)
Neuron
Microglia
Oligo
Endothelia
Astrocyte
1155
542
233
561
21,792
