# Supplementary material for: Enhanced fear memory after social defeat in mice is dependent on interleukin-1 receptor signaling in glutamatergic neurons
Source: Mol Psychiatry. 2024 Mar 8;29(8):2321–34. doi: 10.1038/s41380-024-02456-1 (PMC11412902; doi:10.1038/s41380-024-02456-1)
Supplement: Supplementary file 3 — Supplemental Figure Legend [file 41380_2024_2456_MOESM3_ESM.docx]

**Supplement Figure 1: Single-nuclei RNAseq clustering after RSD.** A) Male IL-1R1^+/+^ and *Vglut2^+^*/IL-1R1^-/-^ (nIL-1R1^-/-^) mice were subjected to RSD or were undisturbed (control) and the hippocampus was dissected, pooled (3 mice per group) and nuclei were collected fourteen hours after RSD. Nucleus RNA profiles were determined by snRNA-seq. B) UMAP clustering from a total of 25,932 nuclei identified 19 unique cell clusters. C) UMAP cluster distribution for each condition: Con-IL-1R1^+/+^, Con-nIL-1R1^-/-^, Stress-IL-1R1^+/+^, Stress-nIL-1R1^-/-^. D) Annotation of each cell cluster based on markers found within the ConservedMarkers function. E) Dot plot shows the expression of cell-specific markers in the 19 clusters. F) Pie Chart represents the Percent of hippocampal nuclei clusters that were neurons, oligodendrocytes, astrocytes, microglia or endothelia based on markers found within the ConservedMarkers function.
